# Artificial Light-Harvesting Nanotubes: Intermolecular Charge Transfer beyond the Exciton Model

**DOI:** 10.1021/jacs.6c17147

**Published:** 2026-10-13

**Authors:** Davide Giavazzi, Anna Painelli

**Affiliations:** † Department of Chemistry, Life Sciences and Environmental Sustainability, University of Parma, Parco Area delle Scienze 17/A, 43124 Parma, Italy

## Abstract

Self-assembled nanotubes of C8S3 cyanine dyes have been widely investigated as model systems for light-harvesting complexes. Recently published data point to a brick-layer molecular arrangement in the nanotubes, in contrast to the herringbone structure previously adopted to explain the absorption and linear dichroism data within the exciton model. Here we solve this conundrum by showing that, relying on the brick-layer structure as extracted from cryo-EM, the exciton model can properly account for the observed spectral features, provided it is extended to account for intermolecular charge-transfer interactions.

## Graphical abstract

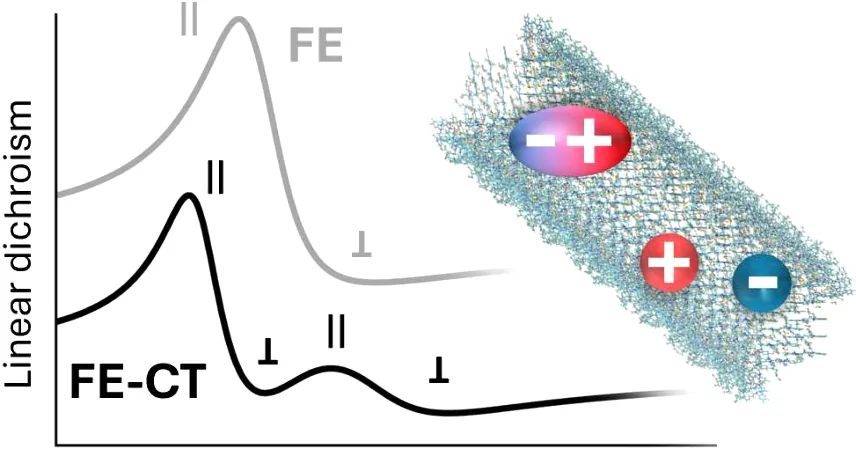

Artificial photosynthetic antennas are convenient model platforms to investigate the mechanisms underlying the efficient exciton harvesting, transport, and concentration observed in natural photosynthetic systems.^1–4^ Aggregates of 3,3′-bis­(2-sulfopropyl)-5,5′,6,6′-tetrachloro-1,1′ -diactyl benzimidacarbocyanine (C8S3, Figure 1a) are ideal systems to investigate how supramolecular organization, disorder, and vibronic coupling determine exciton dynamics and energy transport and are extensively studied in this respect.^5–15^ When dispersed in a water–methanol mixture, C8S3 dyes spontaneously self-assemble into well-defined homogeneous μm-long double-wall nanotubes (DWNTs) with an inner diameter of ∼6 nm and an outer diameter of ∼14 nm.^16–22^ The DWNTs show two main absorption bands, at 598 and 588 nm, ascribed to the inner and outer wall, respectively.^18,20,23,24^ These narrow absorption bands, largely red-shifted versus the monomer (Figure 1b), are a clear signature of extended delocalization in J-aggregates. Quite interestingly, the outer wall of the DWNTs can be selectively removed, maintaining the integrity of the inner wall, the negligible interwall interaction allowing the isolation of the spectrum of the inner wall nanotube (IWNT) from the total absorption spectrum.^23–26^ Measurements on partly aligned samples in a flow cell^24^ show four distinct transitions in the absorption spectrum of the IWNT with two bands polarized along the nanotube axis and two bands perpendicularly polarized (Figure 1c), as confirmed by linear dichroism (LD) (Figure 1d). Relying on the recently published cryo-EM structure of the IWNT,^27^ we demonstrate that these spectral features can be rationalized provided the Frenkel exciton (FE) model is extended to account for intermolecular charge-transfer (CT) interactions, offering a new and much needed interpretative tool to investigate the photophysics of these intriguing systems.

**Figure 1:**
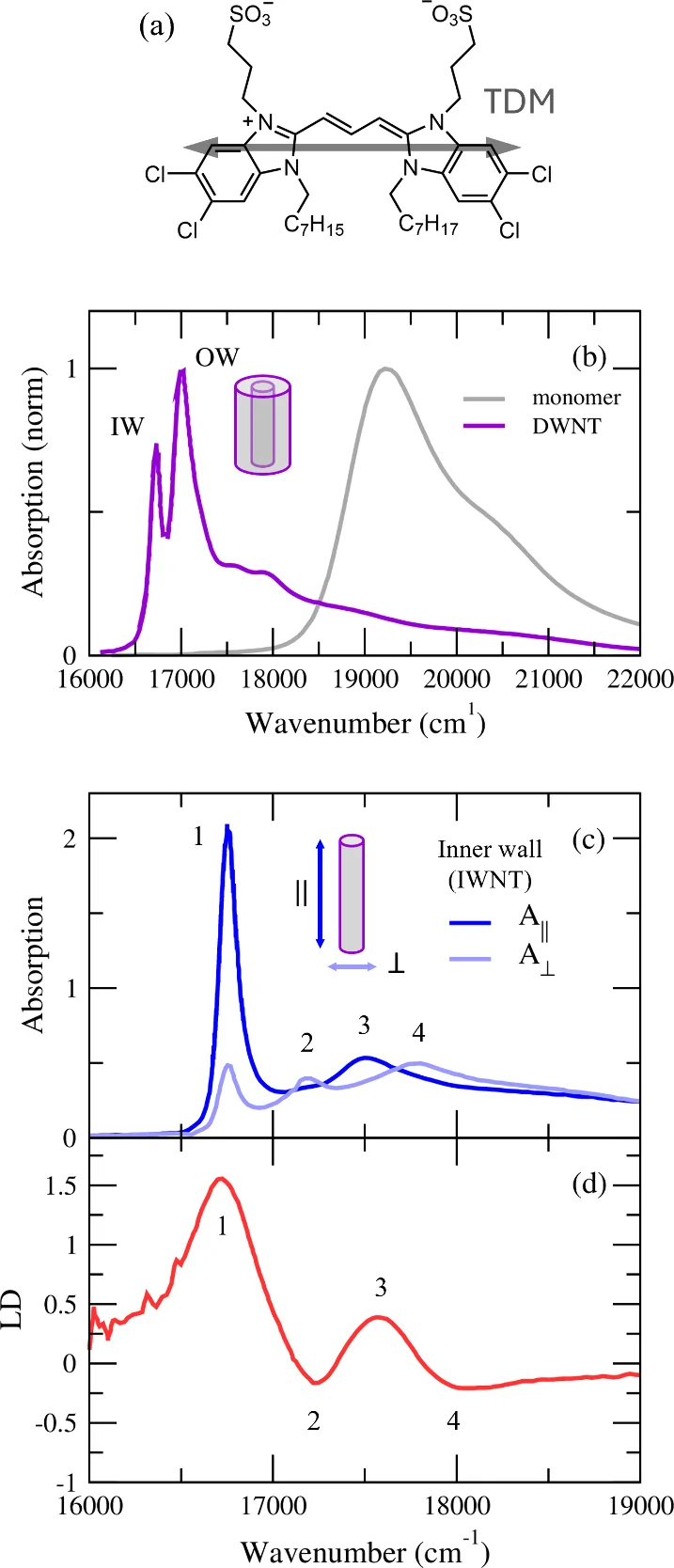
(a) Chemical structure of C8S3. The double sided arrow indicates the orientation of the *S*_0_ → *S*_1_ TDM. (b) Absorption spectrum of the monomer in methanole (gray) and DWNT in a water/methanol mixture (purple). (c) Polarized absorption and (d) linear dichroism spectrum of the IWNT. Reproduced from ref 24. Copyright 2013 American Chemical Society.

A first effective model for the optical spectra of the IWNT was proposed in ref 23. Detailed information on the molecular arrangement inside the nanotube was not available at that time, so a supramolecular structure was guessed to reproduce experimental spectra. Specifically, the four experimentally observed transitions of the IWNT and their polarization were rationalized within the framework of the FE model, i.e., only accounting for electrostatic intermolecular interactions, assuming a 2D herringbone (HB) supramolecular arrangement with two molecules per unit cell wrapped on a cylindrical surface. Extensive Molecular Dynamics (MD) simulations were later performed,^26,28–33^ but the simulation of the complete self-assembly process from solution remains computationally prohibitive, even at the coarse-grained level, due to the large system size and the long time scales involved. Therefore, MD simulations were limited to address the stability of preassembled nanotube models^26,28,29,31^ and to investigate the early stages of the outer-wall formation templated by the inner wall.^32^ While MD results confirmed the stability of the herringbone arrangement, they also suggested a possibly more stable brick-layer (BL) packing.^28,31,32^

Recently, Caram and co-workers obtained the structure of the IWNT at near-atomic resolution via cryo-EM,^27^ unequivocally showing that the C8S3 dyes adopt a slip-stack BL arrangement in a 2D lattice with a single molecule per unit cell wrapped to form a cylinder (see Figure 2a,b and section 1 in SI). The BL packing was further corroborated by polarization-resolved wide-field photoluminescence microscopy measurements performed on individual nanotubes.^34^ Experimental data firmly establish the BL structure for the IWNT, opening a fundamental problem, since with this structure the FE model predicts only two bands in the absorption spectrum, polarized parallel and perpendicular to the tube’s long axis, the latter arising from two degenerate states.^9,11,35–39^ In ref 27 a simplified theoretical model was proposed in which the system was described by effective (2 × 2) Hamiltonians with all possible kinds of intermolecular interactions collapsed in a series of empirical model parameters. However, as pointed out in refs 34 and 40 , a detailed theoretical model properly addressing the absorption and linear dichroism spectra of the IWNT, relying on the experimentally resolved structure, is still missing. Here, we present a comprehensive model that, building upon the experimentally resolved supramolecular structure, quantitatively accounts for the spectral properties of the IWNT in terms of an FE model extended to account for intermolecular CT degrees of freedom (FE-CT model).

**Figure 2:**
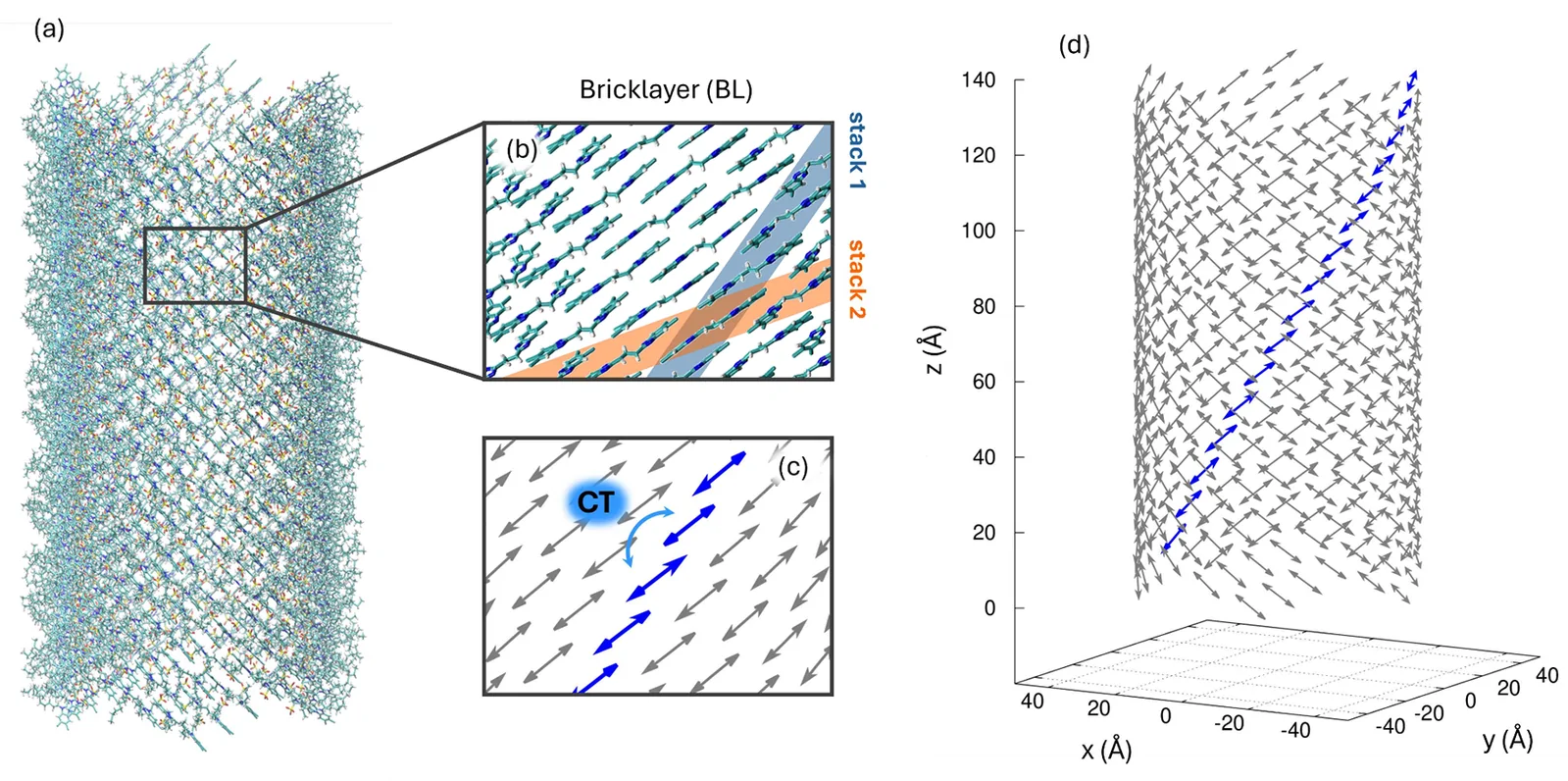
(a) Cryo-EM resolved atomic structure of the IWNT from ref 27. (b) Blow-up of the 2D structure of the nanotube wall (side chains are omitted for clarity). (c) Sketch of a portion of the nanotube wall, representing each dye as a double arrow corresponding to the relevant TDM. (d) Structure of the nanotube reconstructed in terms of TDMs. In panels c and d, the blue arrows mark the dyes belonging to stack 1.

First, we reconstruct the structure of the nanotube in terms of oriented transition dipole moments (TDMs) as shown in Figure 2c,d (see SI section 1 for details). Cryo-EM data show small deformations of the polymethine bridge of each monomer in the aggregate (Figure 2b). Single point TD-DFT calculations on randomly selected molecules extracted from the cryo-EM structure confirm that these distortions do not qualitatively alter the excitation spectrum at the molecular level. Indeed, in all investigated geometries, the *S*_0_ → *S*_1_ transition is delocalized through the π-conjugated core and well separated in energy from *S*_0_ → *S*_2_ (see SI, section 2). In the spirit of the FE model, we then only account for the *S*_0_ → *S*_1_ transition on each molecular unit. Its energy *E*_F_ = 2.34 eV and TDM ≈ 11.4 D are estimated from the experimental absorption spectrum of C8S3 in ethanol solution.^36^ Electrostatic intermolecular interactions between TDMs, the only additional ingredient of the FE model, are introduced in the extended dipole approximation, setting the dipole length at 5.27 Å and accounting for an effective refractive index η = 1.34 to screen the interactions. As already stated, absorption and LD spectra calculated in the FE model show the appearance of a single transition in each of the two polarizations (Figure 3b,c), in contrast with experimental data.

**Figure 3:**
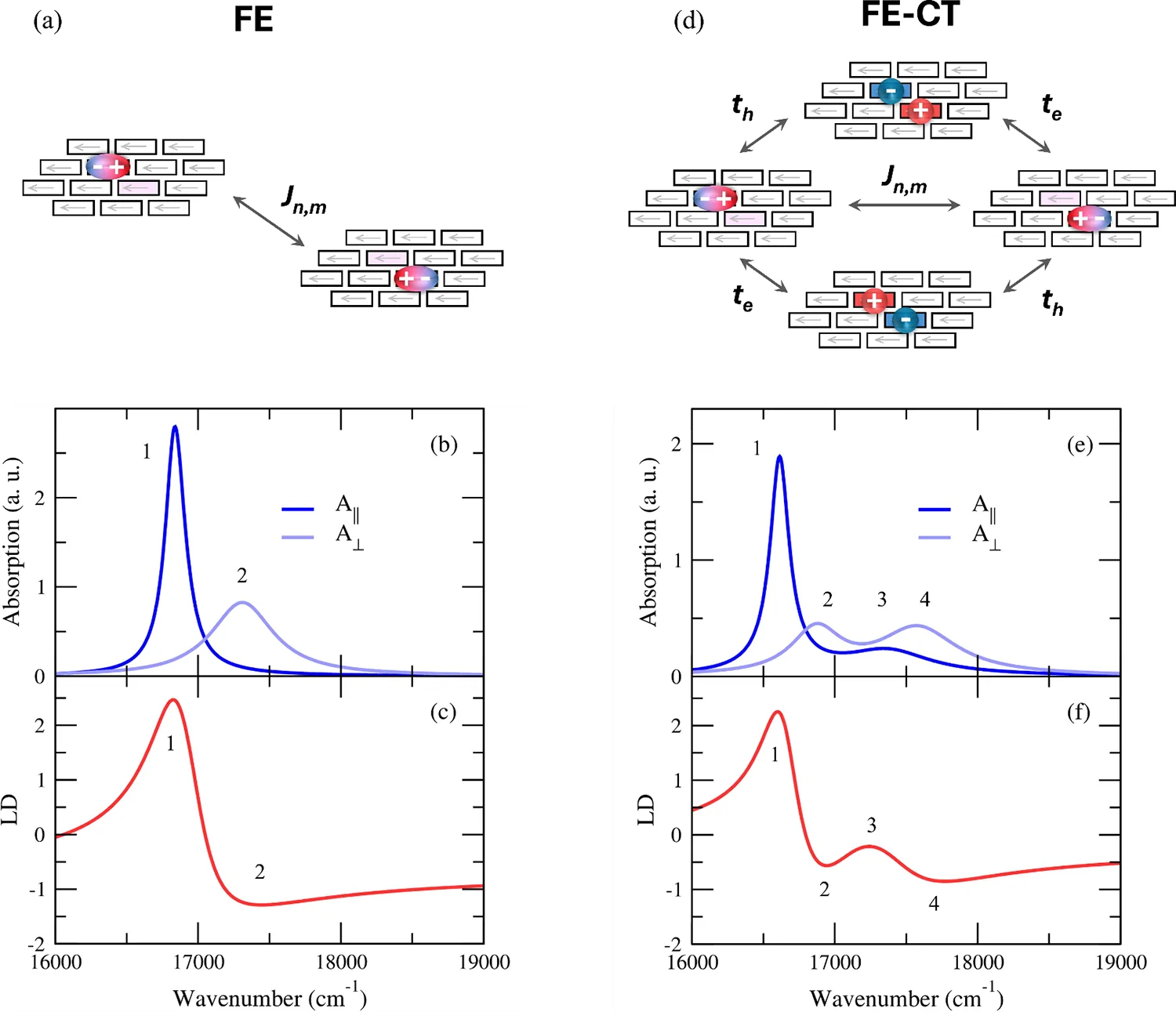
(a) Sketch of the FE model: localized excitons are represented as an electron–hole pair residing on the same site, with the *J*_*nm*_ measuring the probability of exciton hopping between sites *n* and *m*. Panels b and c show the (polarized) absorption and LD spectra calculated in the FE model. (d) Sketch of the FE-CT model: besides localized excitons, as in the FE model, electron and hole hopping are introduced to generate CT states, where a positive and a negative charge are located on nearby sites. Panels e and f show the (polarized) absorption and LD spectra calculated in the FE-CT model. A nanotube of 130 nm (3900 sites) is considered (results for other system sizes in the SI). A Lorentzian line shape of appropriate width is assigned to each transition (see SI).

Although not explicitly investigated, the possible involvement of CT states was already suggested,^11,27^ and more recently, CT interactions have been demonstrated in J-aggregates of a different family of cyanine dyes.^41^ We therefore extend the FE model to include CT interactions.^42–49^ Details can be found in the SI; here we just mention that we only account for electron and hole hopping (*t*_*e*_ and *t*_*h*_, respectively) among nearest-neighbor sites along the directions of a π-stack, marked by shadowed blue (stack 1) and orange areas (stack 2) in Figure 2b (hoppings along other directions are neglected due to the poor spatial overlap between the involved molecular orbitals). Allowing for intermolecular electron/hole hopping considerably extends the basis set to include CT states, i.e., states where molecules with positive and negative charges are present. To reduce the computational effort, we cut the basis to only account for states with a single nearest neighbor CT pair along the considered stack (results for the inclusion of non-nearest neighbor CT are shown in the SI, section 5). The mixing of Frenkel and CT excitons has sizable effects when the two families of states are close in energy. The energy of CT states is calculated as $E_{\mathrm{CT}}^{0}$ plus a correction that accounts for the electron–hole electrostatic attraction. This correction is comparatively small for stack 2, so that relevant CT states lie at high energies and have marginal spectroscopic effects. Accordingly, spectra in Figure 3e,f are calculated only accounting for CT interactions along stack 1 (spectra accounting for both interactions are shown in the SI, section 6). Best results are obtained setting $E_{\mathrm{CT}}^{0}=2.95$ eV and *t*_*e*_ = *t*_*h*_ = 0.015 eV, which, together with the effective refractive index, are the only adjustable model parameters.

Calculated spectra in Figure 3e,f agree well with experimental data in Figure 1c,d, with bands 1 and 3 polarized along the nanotube axis and bands 2 and 4 perpendicularly polarized. The relative intensity of the absorption bands also favorably compares to the experiment. The residual perpendicular polarization of the most intense band in the experimental spectrum in Figure 1c and the minor deviations of calculated and experimental linear dichroism intensities are ascribed to the nonperfect alignment of the nanotubes in the experimental sample. Introducing different sources of static disorder does not significantly alter the results (see SI, section 8), demonstrating that a moderate amount of disorder in the diagonal energies and even a comparatively large disorder on the hopping integrals do not considerably affect the shape of the spectra.

We emphasize that the position and the relative intensity of the absorption bands depend on the specific choice of model parameters. However, once the supramolecular structure is fixed, the number of the absorption bands and their polarization are an intrinsic feature of the adopted model. In the experimental BL structure, the FE model cannot originate four distinct transitions regardless of the choice of model parameters. Having ruled out a splitting of the electronic excitation at the molecular level and the possible role of vibronic coupling (see SI, section 9), the inclusion of CT states represents the only viable mechanism to simulate the optical spectra of the IWNT. CT states in our basis set are fully disconnected from the ground state and are intrinsically optically dark; however, the electron/hole hopping allows for their hybridization with Frenkel excitons from which they borrow intensity, effectively doubling the number of absorption bands, as schematically illustrated in Figure 4. It is important to stress that under these conditions photoexcitation does not generate free charges nor pure CT states, but hybrid states with a mixed Frenkel–CT character.

**Figure 4:**
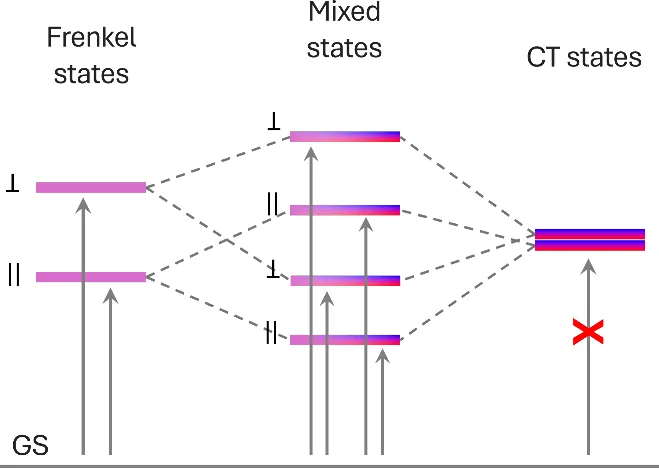
Graphical representation highlighting the mixing between Frenkel excitons and CT states. The arrows mark optically allowed transitions.

As a final topic, we briefly discuss the fluorescence behavior of aggregates. Experimental values of quantum yield and fluorescence lifetime for the DWNT^9,10,12,13,25,30,50,51^ suggest a moderate change of the radiative lifetime upon aggregation (see section 11 in the SI), which is hard to reconcile with the super-radiant nature of the highly delocalized J aggregates in the nanotube.^30,52^ The proposed presence of a low-lying dark excited state below the super-radiant state^15^ could solve the issue, leading however to a decreasing fluorescence quantum yield with decreasing temperature. In our model, the lowest excited state is bright and super-radiant (section 12 in SI) therefore we propose an alternative tentative explanation. The large delocalization of the exciton in the nanotube originates a very dense manifold of dark states just above the lowest bright state. In the reasonable hypothesis that thermalization among these densely distributed states is faster than radiative and nonradiative decays, emission occurs from the thermally equilibrated exciton manifold with a reduced population of the lowest-energy super-radiant state, thus explaining the observed moderate fluorescence quantum yield. This interpretation is consistent with the observed increase in the quantum yield with decreasing temperature.^51^

In conclusion, starting from the atomically resolved cryo-EM structure for the C8S3 IWNT,^27^ a comprehensive model is proposed to address the optical spectra of this widely investigated artificial light-harvesting system, demonstrating that the observed spectral features can only be described by extending the FE model, which describes a system where electrons are fully localized in each molecular unit, to account for electron hopping between adjacent sites, effectively introducing a mixing between Frenkel and CT excitons.

## Supplementary Material



## Notes

Stuctural model, detailed description of the model Hamiltonian, and additional computational results (PDF)
