# Supplementary material for: Artificial Light-Harvesting Nanotubes: Intermolecular Charge Transfer beyond the Exciton Model
Source: J Am Chem Soc. 2026 Oct 13;148(42):45308–13. doi: 10.1021/jacs.6c17147 (PMC13721161; doi:10.1021/jacs.6c17147)
Supplement: Supplementary file 1 [file ja6c17147_si_001.pdf]

Supporting Information

Artificial Light-Harvesting Nanotubes: Intermolecular Charge Transfer Beyond the Exciton Model

Davide Giavazzi[1] and Anna Painelli*[1]

[1]Department of Chemistry, Life Sciences and Environmental Sustainability, University of Parma, Parco Area delle Scienze 17/A, 43124 Parma, Italy

*Email: anna.painelli@unipr.it

Contents

1 Nanotube structure **2**

2 Single point calculations **4**

3 FE-CT Hamiltonian **5**

4 Calculation of absorption and linear dichroism **7**

5 Non-nearest-neighbor CT states **8**

6 CT states along stack 1 and 2 **9**

7 Size convergence **10**

8 Static disorder **10**

9 Vibronic coupling **12**

10 CT contribution to the eigenstates **13**

11 Fluorescent behavior of the DWNT **14**

12 Relative sign of t_e and t_h and dark states **15**

1 Nanotube structure

We reconstruct the inner wall nanotube (IWNT) structure as an ordered array of oriented transition dipole moments (TDMs) that faithfully reproduces the molecular arrangement resolved by cryo-EM by Caram and co-workers in ref.[1]. Each TDM is oriented along the π-conjugated backbone of the chromophore. To define the dipole orientation we consider the vector connecting the two terminal carbon atoms of the polymethine bridge. The experimental structure exhibits a small degree of conformational disorder, with individual molecules adopting slightly different geometries. At first we get rid of structural disorder averaging the orientations of a random selection of molecules. An oriented dipole is placed on a ring of radius 36.7 Å on the xy plane and is replicated by applying a rotation of $\theta = 19.2°$ about the ring axis together with a translation of $h = 4.95$ Å along z to form a helix (Figure S1a, b). The helix is then replicated twice by applying a rotation of $\theta = 11.2°$ and a traslation of $h = -3.3$ Å to obtain a stripe (Figure S1c), and finally the stripe is replicated four times through successive rotations of $\theta = 72°$ to obtain a cylindrical surface homogeneously covered with a bricklayer (BL) motif with one molecule per unit cell (Figure S1d).

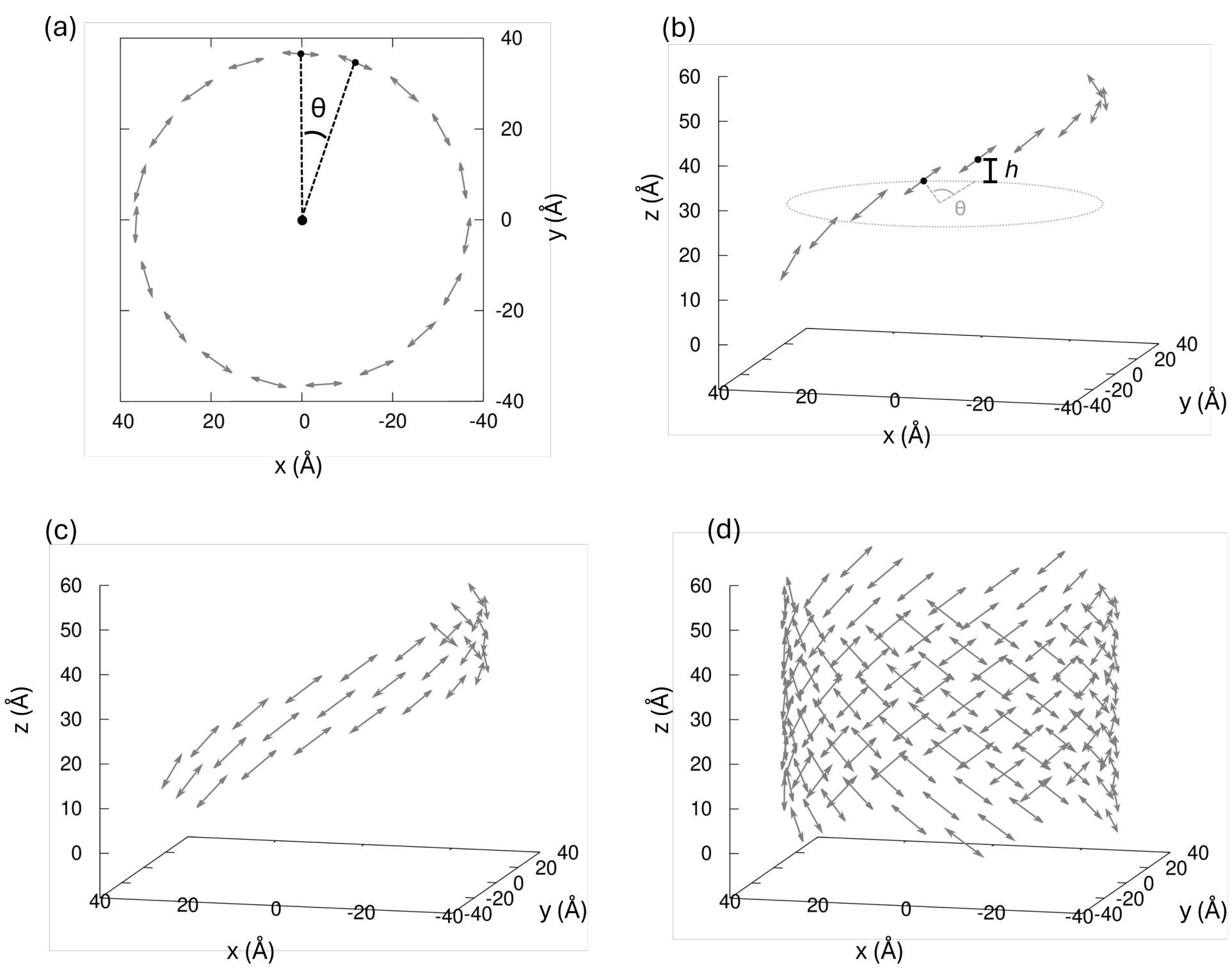

Figure S1: Reconstruction of the cryo-EM resolved structure of the inner wall of C8S3 nanotubes. Experimental structure from ref.[1]. Gray double arrows represent the oriented molecular transition dipole moments.

Figure. S2 shows how the 2D BL layer wraps into a cylindrical shape with C5 symmetry around the IWNT axis and 6 molecules in the unit cell.

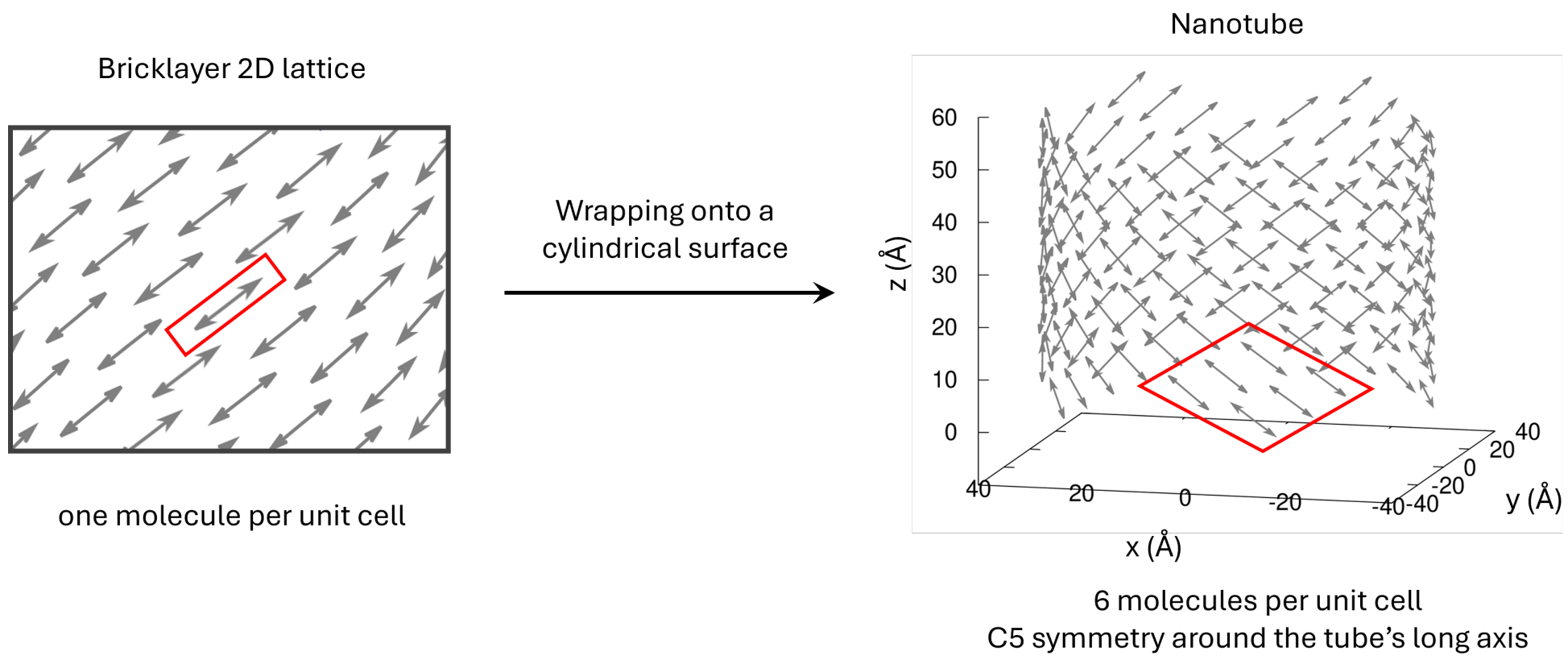

Figure S2: The simple BL 2D structure with one molecule per unit cell wraps up into a cylindrical shape with C5 symmetry around the IWNT axis and 6 molecules per unit cell (unit cells indicated with the red boxes).

2 Single point calculations

In cyanine dyes, the first singlet excited state S_1 is typically well separated in energy from S_2 and higher-lying excited states. As a result, the Heitler–London approximation (i.e., the inclusion in the aggregate Hamiltonian of only the local $S_0 \rightarrow S_1$ excitation) provides an accurate description of the exciton manifold. In systems where S_1 and S_2 are close in energy, this approximation breaks down and both states have to be included in the Hamiltonian, typically giving rise to multiple sets of transitions in the absorption spectrum. As shown in Figure 2b in the main text, the chromophoric core of C8S3 molecules in the nanotube is slightly distorted. In principle, this distortion could reduce the delocalization of S_1 and lead to the formation of two nearly degenerate states localized on the benzimidazole units, making the HL approximation inapplicable and giving rise to the experimentally observed doubling of the spectroscopic features.

To test this hypothesis we performed gas-phase single point TD-DFT calculations (CAM-B3LYP/6-31++G(d,p)) on a random selection of geometries extracted from the cryo-EM structure. The results in Table S1 show that for all the investigated geometries, S_1 remains bright and well separated in energy form the nearly dark S_2. The slight distortion of the polymethine bridge therefore does not qualitatively modify the electronic structure of the optimized, undistorted chromophore, ruling out the involvement of S_2 and higher local excitation as the origin for the observed spectroscopic features.

Molecule	$S_0 \rightarrow S_1$ (eV)	f_{01}	$S_0 \rightarrow S_2$ (eV)	f_{02}
optimized	3.0826	1. 9869	4.2549	0.0000
1	2.6421	1.0249	3.9817	0.0433
2	2.6650	1.2489	4.2675	0.1150
3	2.6633	1.0144	3.9856	0.0354
4	2.5785	1.1972	4.2791	0.1709
5	2.6268	1.0455	4.0259	0.0588
6	2.6442	1.0873	3.9890	0.0312
7	2.6491	0.9950	3.9618	0.0288
8	2.5393	1.1772	4.3008	0.1654
9	2.6480	1.0987	4.0809	0.0684
10	2.6486	1.0085	3.9358	0.0237

Table S1: Energies and oscillator strengths (f) of S_1 and S_2 for the optimized chromophore and for 10 conformations extracted from the experimental structure from ref.[1]. Lateral alkyl side chains are cut and replaced by hydrogen atoms. Gas-phase single point TD-DFT calculations performed at the CAM-B3LYP/6-31++G(d,p) level of theory.

3 FE-CT Hamiltonian

We indicate as $|n, m\rangle$ a state with a hole on site n and an electron on site m (we only consider states with a single electron-hole pair), with a local Frenkel excitation defined by an electron and a hole residing on the same site ($|n, n\rangle$). The complete FE-CT Hamiltonian reads:

$$\begin{aligned}
\hat{H} = E_F \sum_n |n, n\rangle\langle n, n| + \sum_{n,m\neq n} J_{m,n}|m, m\rangle\langle n, n| \\
+ \sum_{n,m\neq n} E_{CT}(n, m)|n, m\rangle\langle n, m| \\
+ \sum_{n,m}\sum_s t_e(s)\,[|n, m\rangle\langle n, m + s| + h.c.] \\
+ \sum_{n,m}\sum_s t_h(s)\,[|n, m\rangle\langle n + s, m| + h.c.]
\end{aligned} \tag{S1}$$

where $s(\neq 0)$ defines the distance between two sites. The first two terms in the above equation describe the FE model, with E_F measuring the site energy of the molecular excitation and $J_{m,n}$ measuring the electrostatic intermolecular interaction between sites m and n. The subsequent terms account for CT interactions. Specifically, the energy of the charge transfer states $E_{CT}(n, m)$ is defined as:

$$E_{CT}(n, m) = E^0_{CT} - \frac{e^2}{4\pi\varepsilon_0\eta^2 d_{nm}} \tag{S2}$$

where E^0_{CT} is kept as a free parameter and the second term accounts for the electrostatic interaction between the electron and hole, d_{nm} being the distance between sites n and m and η the medium refractive index. The last two terms account for electron and hole hopping. Specifically, the third (fourth) line describes the hopping of an electron (hole) from site m (n) to site $m + s$ ($n + s$), governed by the electron (hole) hopping integral $t_e(s)$ ($t_h(s)$), while the hole (electron) remains fixed on site n (m). Given the short-range nature of the hopping integrals, we only consider $t_{e(h)} \neq 0$ when m (n) and $m + s$ ($n + s$) are nearest-neighbor sites along the direction of a π-stack (CT interactions along other directions are neglected due to the poor spatial overlap between the involved molecular orbitals).

The nearest-neighbor distance along stack 2 (d_{nm} = 13.17 Å) is fairly large, leading to a small electron-hole electrostatic interaction -0.607 eV (setting the screening $\eta^2 = 1.8$). Therefore, CT states along stack 2 are located to fairly large energies if compared to CT states along stack 1, where a shorter nearest-neighbor distance d_{nm} = 9.7 Å gives a larger stabilizing electrostatic interaction of -0.825 eV. CT states along stack 2 then prove irrelevant for the photophysics in the investigated spectral region, even in presence of sizable hopping integrals, and we therefore exclude them from the Hamiltonian. Results for explicit inclusion of CT

along both stacks can be found in the following sections.

The dimension of the basis set grows very rapidly with the number of sites, we therefore introduce a further approximation by restricting the electron and hole to nearest-neighbor sites along the considered stack. Results for the inclusion of non-nearest neighbor CT states can be found in the following.

Electrostatic intermolecular couplings $J_{m,n}$ are evaluated as the Coulomb interaction between TDMs on site n and m, where each TDM is described as an extended dipole with a length of 5.27 Å and two opposite charges of $\pm 0.45e$ (as to regain the C8S3 experimental TDM of 11.4 D) centered in the site position and oriented according to the structure shown in Figure S1 and Figure 2 in the main text. On-site exciton energy is fixed to $E_F = 2.34$ eV (taken from the position of the maximum in the absorption spectrum of the monomer in solution). E_{CT}^0 and the hopping integrals are fitting parameters and their values are fixed to $E_{CT}^0 = 2.95$ eV and $t_e = t_h = 0.015$ eV. As better detailed in section 11, the two hopping integrals actually define a single parameter relevant to the absorption spectrum which is their sum $|t_e + t_h| = 0.03$ eV.[2]

4 Calculation of absorption and linear dichroism

To calculate absorption and LD spectra we define the dipole moment operator as:

$$\hat{\vec{M}} = \sum_n \vec{\mu}_n \{|G\rangle\langle n,n| + |n,n\rangle\langle G|\} \tag{S3}$$

where $\hat{\mu}_n$ is the TDM of the n-th molecule, $|n,n\rangle$ is the local Frenkel exciton and $|G\rangle$ is the aggregate ground state, i. e., the state where all molecules are in their ground state. Absorption along x, y and z is calculated as:

$$A_x(\omega) = \sum_i \omega_i |\langle G|\hat{\vec{M}}_x|i\rangle|^2 \frac{\gamma}{(\omega - \omega_i)^2 + \gamma^2} \tag{S4}$$

where $|i\rangle$ are the eigenstates of the Hamiltonian, ω_i being their energies. A Lorentzian bandshape with HWHM γ is assigned to each eigenstate. To better fit the experimental data, an appropriate width is assigned to each eigenstate belonging to one of the four observed bands in the absorption spectrum. Specifically, we respectively assign $\gamma = 0.01$, 0.025, 0.04 and 0.045 eV to band 1, 2, 3 and 4, ordered by increasing energy ($\gamma = 0.01$ and 0.03 eV are used for the two bands in Figure 3b,c, when CT are neglected).

Parallel and perpendicular components of the absorption spectrum for a sample of nanotubes oriented along z are calculated as $A_{||} = A_z$ and $A_\perp = \frac{1}{2}(A_x + A_y)$, the total absorption being $A_{||} + A_\perp$.

Following ref.[3], we define the linear dichroism as:

$$LD = \frac{3(A_{||} - A_\perp)}{A_{||} + 2A_\perp}. \tag{S5}$$

In this way, LD ranges from 3 when $A_\perp = 0$ to -1.5 when $A_{||} = 0$. Smaller experimental LD values with respect to the calculated ones are due to the non perfect alignment of the sample in the experiment.

5 Non-nearest-neighbor CT states

As mentioned in section 3, we include in the Hamiltonian only CT states where the electron and hole reside on nearest-neighbor sites (as the one depicted in Figure S3b). We test the validity of this approximation on a smaller model system. Figure S3a compares the absorption spectrum of a single helix of 52 molecules calculated only accounting for nearest-neighbor CT states (red) and including all possible CT states (green). The two spectra are almost superimposed: the smaller electrostatic interaction between the electron and the hole sets the non-nearest-neighbor CT states at higher energies, making them irrelevant for the low energy photophysics. These states can therefore be safely neglected.

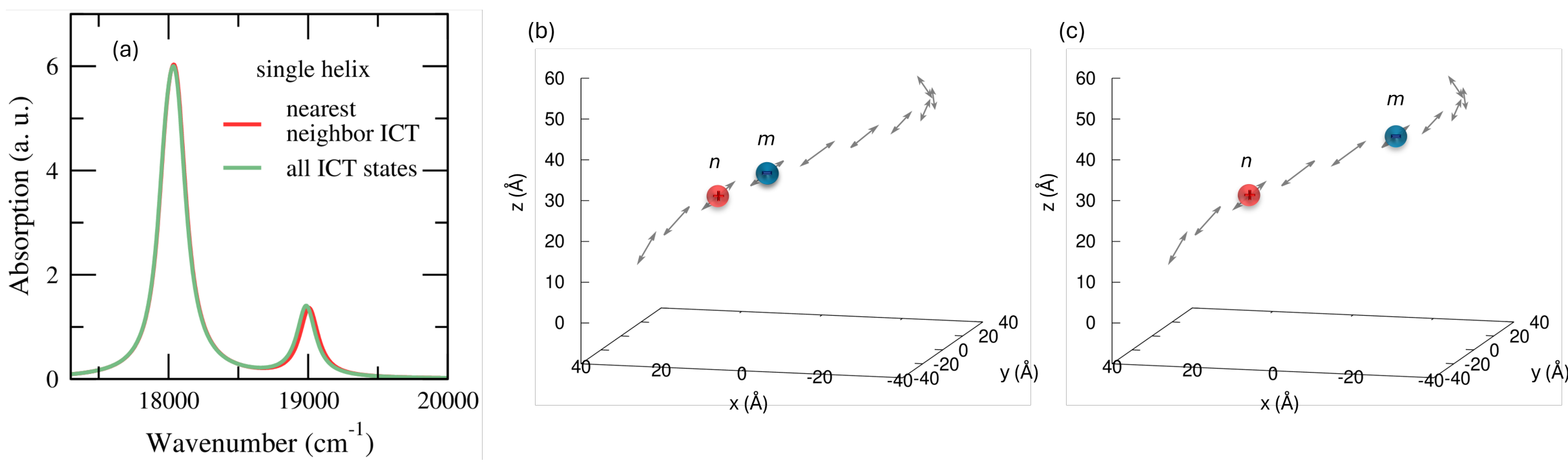

Figure S3: (a) Absorption spectrum for a single helix of 52 molecules calculated according to Eq. S6 including only CT states with nearest-neighbor charges (red) and all possible CT states (green). Sketch of a state with nearest-neighbor (b) and non-nearest-neighbor (c) charges.

6 CT states along stack 1 and 2

As shown in figure S4a,b,c and Figure 2b of the main text, two π-stacks are present in the 2D bricklayer arrangement. CT states along stack 2 lie at higher energies with respect to stack 1 due to the larger nearest-neighbor site distance (see section 3). We therefore account for CT interactions only along stack 1. Here we test this approximation by explicitly including in the Hamiltonian CT interactions along both stacks. We always restrict the basis set to nearest-neighbor CT states and we use the same hopping integrals $t_e = t_h = 0.015$ eV along both directions. Figure S4d,e shows how, even for comparable hopping integrals, CT interactions along stack 2 have a negligible effect on the optical properties of the system.

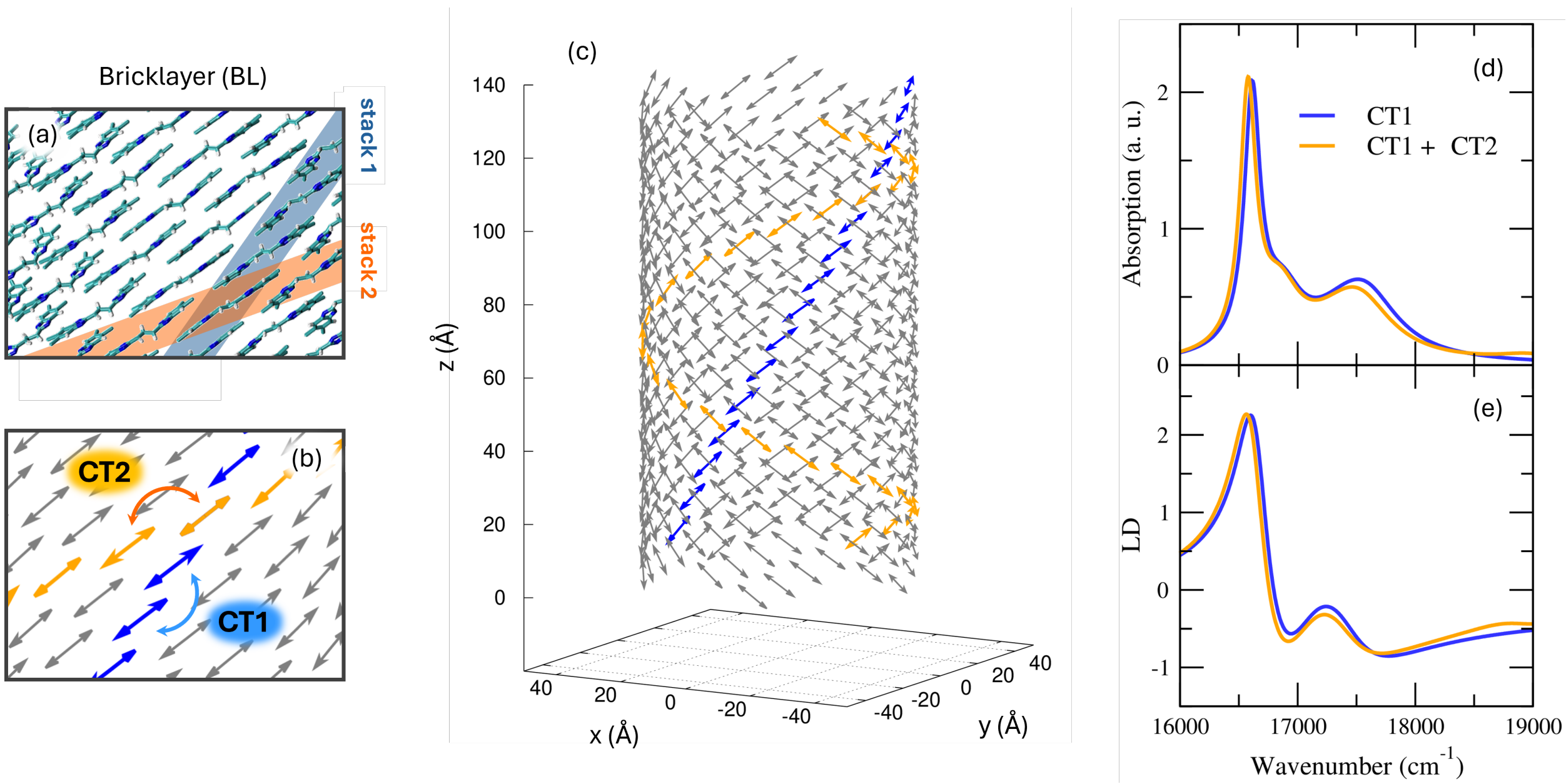

Figure S4: (a) Blow-up of the cryo-EM resolved atomic structure of the inner wall from ref.[1] (side chains are omitted for clarity). Shaded areas highlight the presence of two π-stack motifs. Blow-up (b) and entire structure (c) of the IWNT reconstructed as oriented dipoles with blue and yellow arrows marking the dyes belonging to stack 1 and 2, respectively. Absorption (d) and LD (e) spectra of a tube of 3900 sites calculated according to Eq. S6 including CT along stack 1 (blue) and along both stack 1 and 2 (orange).

7 Size convergence

Unless otherwise specified, we show results for an IWNT of 3900 sites (130 nm). Figure S5 shows the convergence of the results with the size of the system.

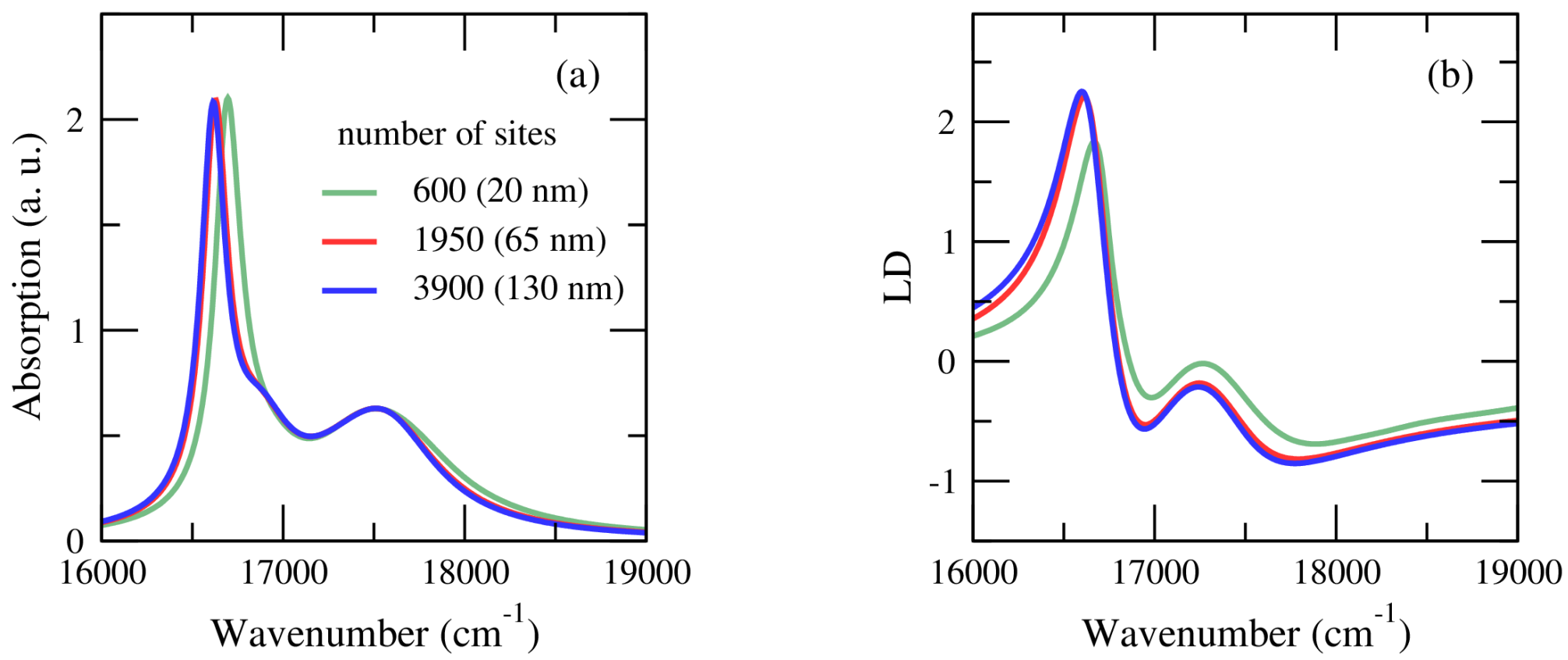

Figure S5: Absorption (a) and LD (b) spectra calculated for IWNTs of different size (as per legend).

8 Static disorder

In the main text, we show results for a perfectly ordered cylindrical lattice. Here we explore the effect of introducing different sources of static disorder.

At first, we introduce diagonal static disorder by adding to the site energies E_F and to the energies of CT states E_{CT} a random fluctuation extracted from a normalized gaussian energy distribution with standard deviation σ. Figure S6 shows how the system is robust up to moderate degrees of disorder. For $\sigma = 0.02$ eV the shape of the absorption spectrum starts to change, possibly indicating a disruption of the delocalized excitons.

Frenkel-CT couplings t_e and t_h are notoriously sensitive to wavefuncion overlap and hence to even sub-angstrom changes in the supramolecular arrangement.[2] Structural disorder can therefore induce significant fluctuations in the values of t_e and t_h. We test our results in this respect by adding to each t_e and t_h a random fluctuation ranging from $\pm\delta$. Figure S7 shows how the shape of the spectra is not really affected by the presence of even very large fluctuations compared to the fixed t_e and t_h values of 0.015 eV.

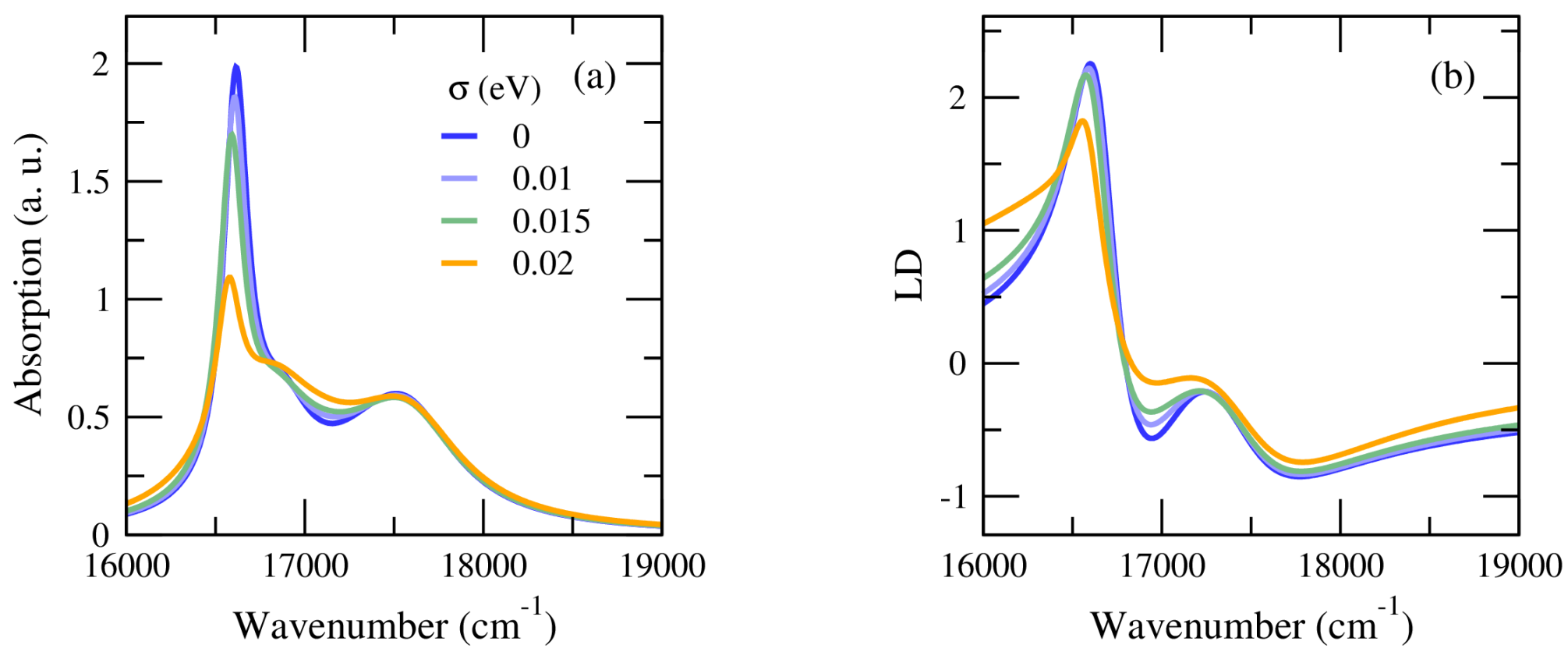

Figure S6: Absorption (a) and LD (b) spectra calculated for IWNTs with different degrees of random gaussian static disorder on the diagonal site energies of Frenkel and CT states.

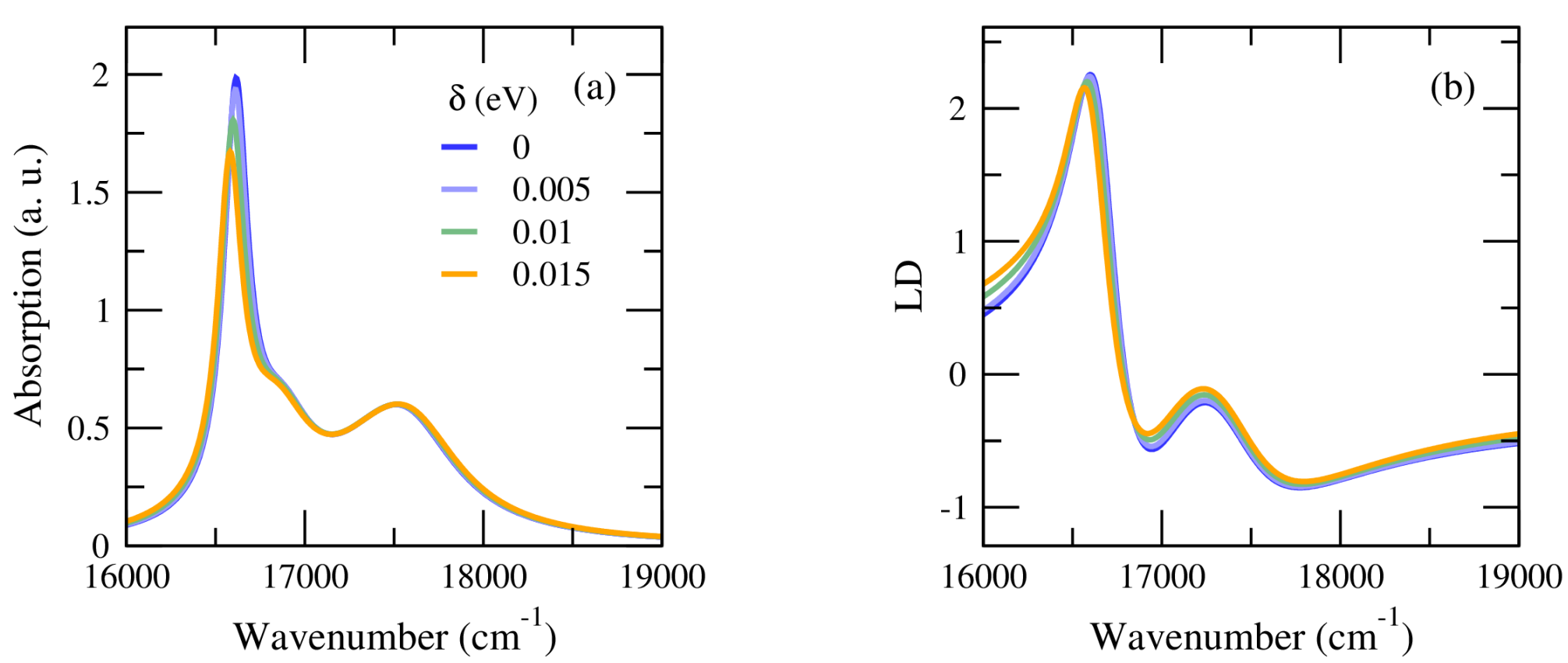

Figure S7: Absorption (a) and LD (b) spectra calculated for IWNTs with different degrees of random static disorder on the hopping integrals t_e and t_h.

9 Vibronic coupling

The absorption spectrum of the monomer in solution (Figure 1b in the main text) shows a clear shoulder $\sim$1200 cm^{-1} to the blue of the main peak, indicating a finite degree of vibronic coupling. However, we exclude that vibronic coupling can be responsible for the multiple features observed in the absorption spectrum of the nanotube. Vibronic coupling notoriously washes out in J aggregates.[4] Moreover, the spacing between the two peaks in the || and $\perp$ spectra of $\sim$750 cm^{-1} and $\sim$600 cm^{-1}, respectively, is not compatible with the typical spacing in vibronic progressions of π-conjugated chromophores. To definitively prove this point, we calculate the absorption spectrum accounting for linear vibronic coupling. The relevant Frenkel-Holstein Hamiltonian reads:

$$\begin{aligned}\hat{H} = E_F \sum_n |n\rangle\langle n| + \sum_{n,m\neq n} J_{m,n} |m\rangle\langle n| \\ + \omega_{vib} \sum_n [\lambda(b_n^\dagger + b_n) + \lambda^2]|n\rangle\langle n| + \omega_{vib} \sum_n b_n^\dagger b_n\end{aligned} \tag{S6}$$

where we used the standard notation $|n\rangle$ for a local excitation on chromophore n, $b_n^\dagger$ and b_n are the bosonic creation and annihilation operators, respectively, for the S_0 harmonic potential of site n, λ^2 is the Huang-Rhys (HR) factor and ω_{vib} the vibrational frequency. We use $\lambda^2 = 0.4$ and $\omega_{vib} = 1200$ cm^{-1}, as estimated from the monomer absorption spectrum.

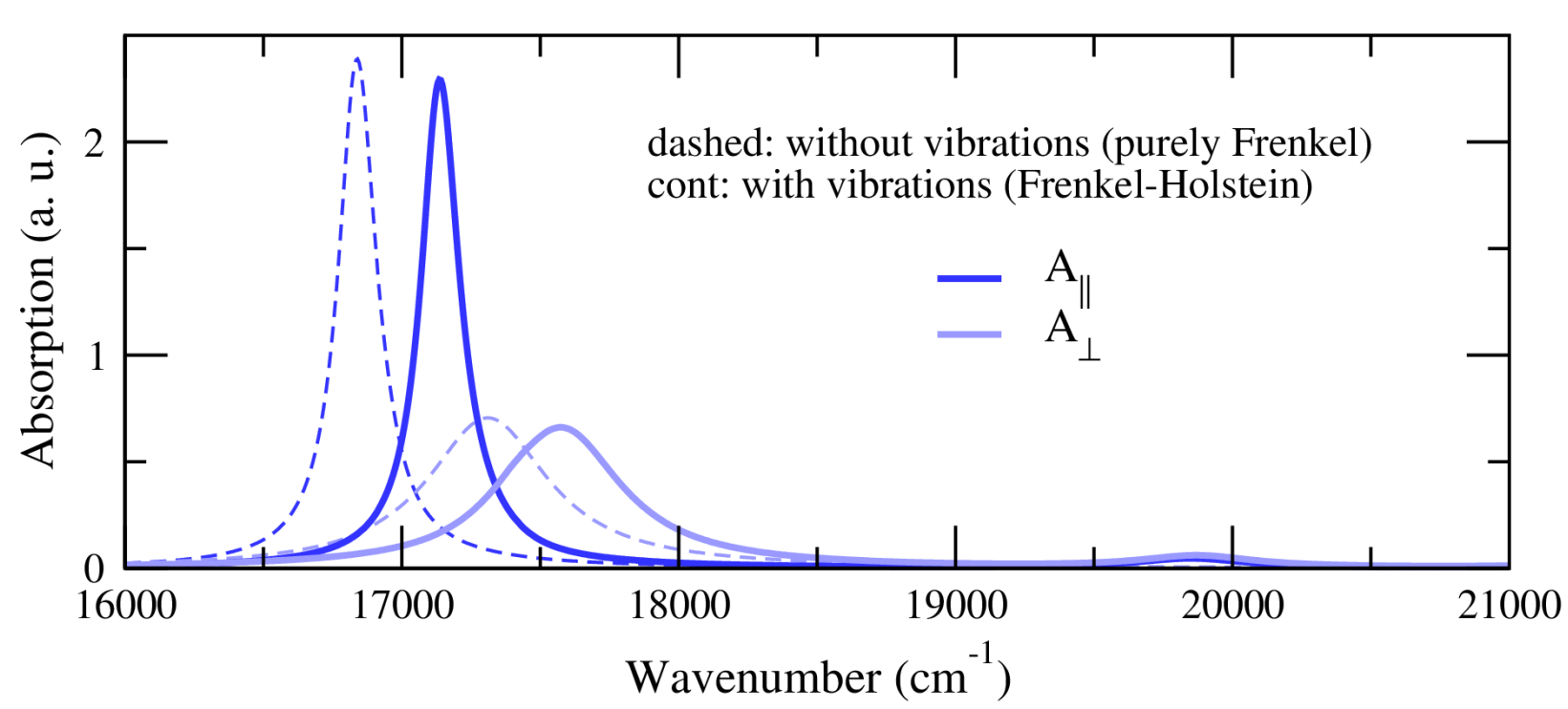

Figure S8: Polarized absorption spectra of the IWNT calculated with the purely Frenkel Hamiltonian (dashed lines) and with the Frenkel-Holstein Hamiltonian (continuous lines) with $\lambda^2 = 0.4$ and $\omega_{vib} = 1200$ cm^{-1}.

Apart from a minor blue-shift of the absorption band, due to the renormalization of the excitonic bandwidth according to the HR factor,[4] Figure S8 shows that the shape and polarization of the absorption spectrum is basically not affected by vibronic coupling. Indeed, in Figure S8 only very weak signatures of vibronic progression are visible around 20000 cm^{-1}, as expected for highly delocalized J aggregates. In the relevant spectral region around 16000 - 19000 cm^{-1} only the two transitions predicted by the FE model are present. Therefore, the appearance of 4 absorption bands in the IWNT spectrum cannot be ascribed to vibronic coupling.

10 CT contribution to the eigenstates

After diagonalization of the Hamiltonian in Eq. S6, we can evaluate the Frenkel and CT contribution to each eigenstate as:

$$F_i = \sum_n |\langle n, n|i\rangle|^2 \tag{S7}$$

$$CT_i = \sum_{n,m \neq n} |\langle n, m|i\rangle|^2 \tag{S8}$$

$|i\rangle$ being the i-th eigenstate. The two contributions clearly sum up to 1. CT contributions for all eigenstates are reported in Figure S9, together with the relevant squared transition dipole moments along the long nanotube axis (μ_z^2) and in the perpendicular direction ($\mu_x^2 + \mu_y^2$).

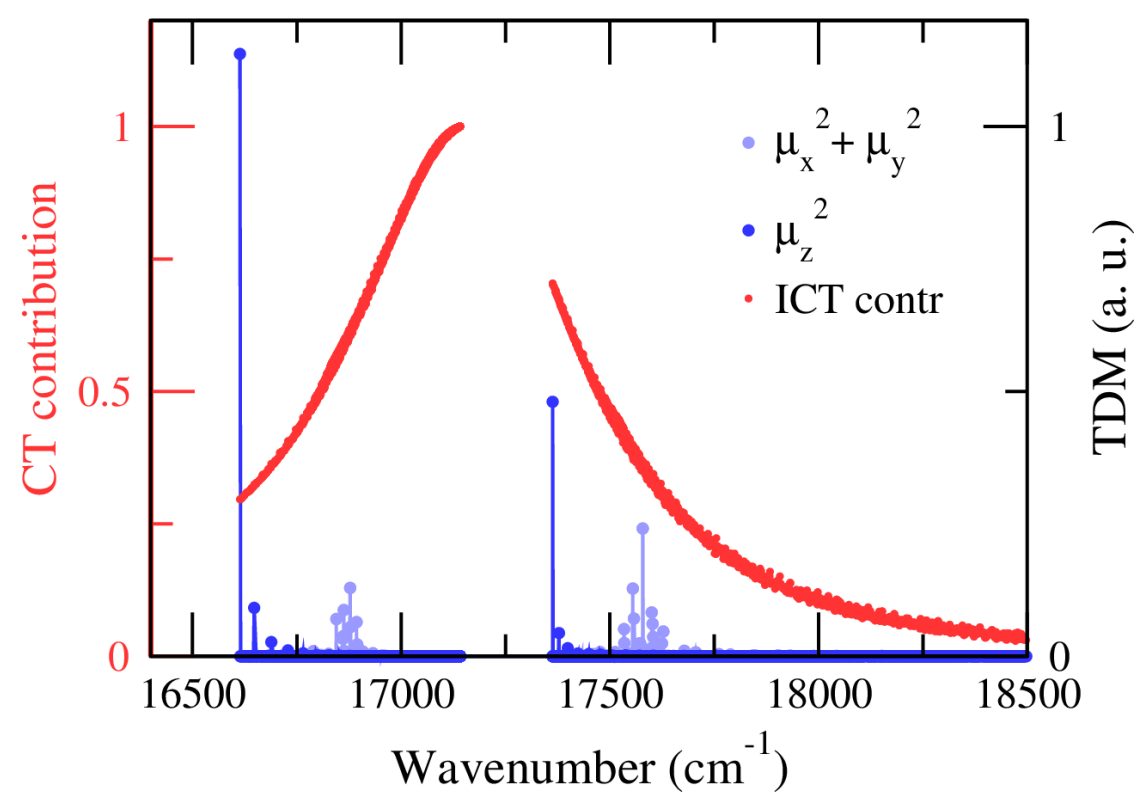

Figure S9: CT contributions and oscillator strengths of the eigenstates of the IWNT.

11 Fluorescent behavior of the DWNT

The quantum yield Φ is related to the radiative and fluorescence lifetimes, τ_R and τ_F, respectively, by the well known expression:

$$\Phi = \frac{\tau_F}{\tau_R} \tag{S9}$$

Upon J-aggregation, the radiative lifetime is expected to decrease considerably: $\tau_R = \tau_{R,m}/\alpha$, with $\alpha >> 1$ in superradiant systems (the m subscript refers to the monomer). Eq. Eq.s S9 and its analogue for the monomer lead to:

$$\alpha = \frac{\Phi}{\Phi_m} \cdot \frac{\tau_{F,m}}{\tau_F} \tag{S10}$$

The fluorescence quantum yield and fluorescence lifetime of the monomer are $\Phi_m \sim 4\%$ and $\tau_{F,m} \sim 110$ ps,[5, 6] while for the DWNT $\Phi_m \sim 3.4\%$ and $\tau_{F,m} \sim 43$ ps.[5, 7], leading to $\alpha \sim 2.17$, a very low value for a highly delocalized J-aggregate. We underline that the fluorescence lifetime accounts for both radiative and non-radiative decay pathways, so the above estimate holds true irrespective of the presence of different non-radiative decay rates in the monomer and the aggregate. Some degree of variability in the experimental values of Φ and τ_F is present in the literature,[8–13] most likely due to different solvation/dispersion environments and experimental setup parameters. Nevertheless, the values always point to fairly low α values.

We therefore propose a tentative explanation to reconcile the experimental lifetimes and quantum yields with the superradiant nature of the aggregate. The exceptionally large exciton delocalization in the nanotube, is responsible for a very dense manifold of dark states (made even more dense due to the presence of CT states) above the lowest bright superradiant state. We assume that thermal equilibrium is reached before any other process takes place. Therefore, emission occurs from a superradiant state with a reduced population ρ, thus reducing the observed quantum yield as $\Phi = \rho\frac{\tau_F}{\tau_R}$. This interpretation is consistent with the observed increase of the quantum yield with decreasing temperature.[11]

12 Relative sign of t_e and t_h and dark states

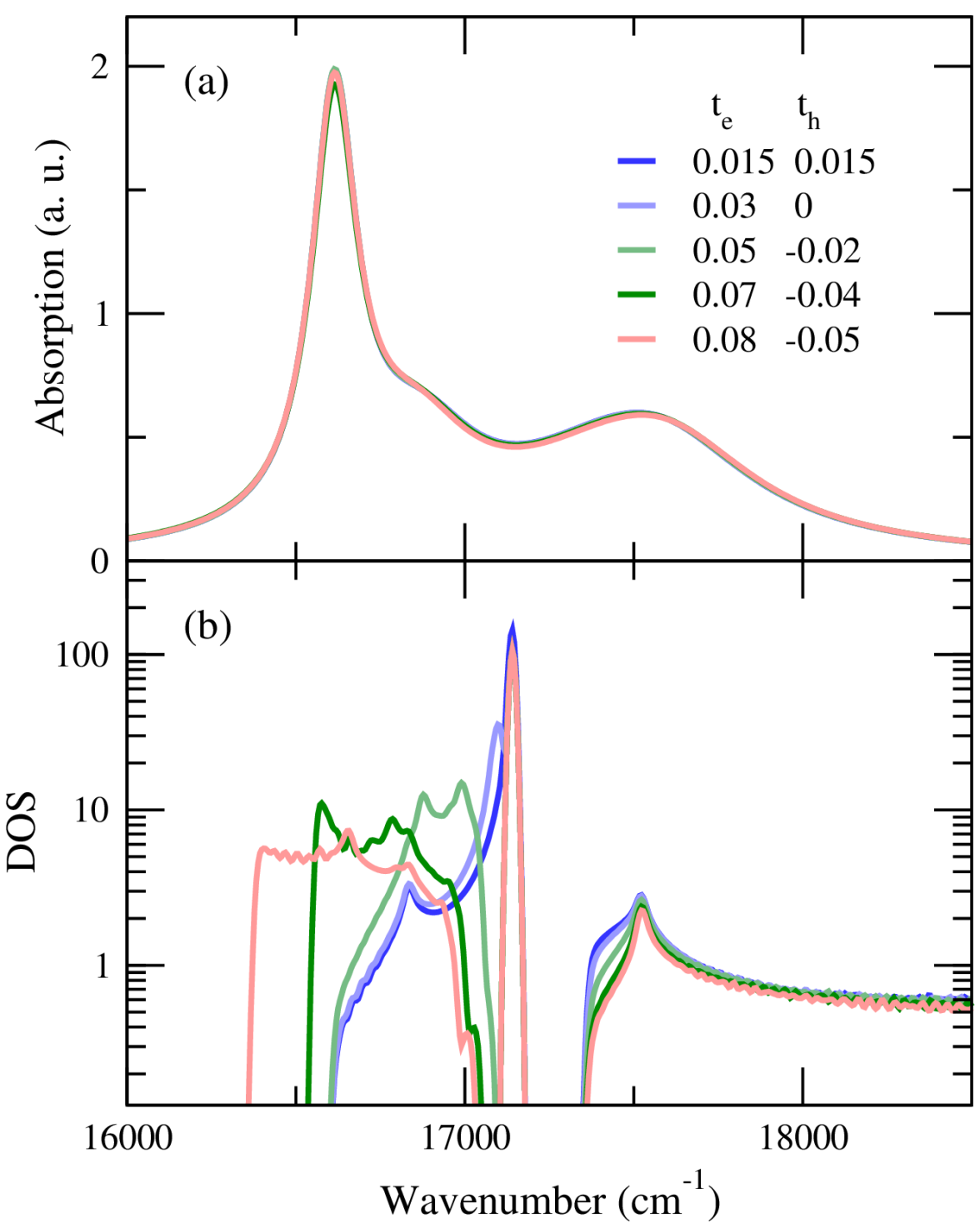

Figure S10: Absorption (a) and density of states DOS (b) calculated for IWNTs with different t_e and t_h (as per legend).

For a periodic lattice, the Hamiltonian in Eq. S6 can be re-written on the basis symmetrized with respect to translation to obtain N independent k-dependent Hamiltonians (N being the number of sites and k being the wavevector). All optically active states belong to the $k = 0$ subspace, in which Frenkel-CT mixing only depends on $|t_e + t_h|$.[2, 4] The sum of the hopping integrals is therefore the only relevant parameter for the calculation of the absorption spectrum, while their difference determines the position of the dark $k \neq 0$ states. This is clearly illustrated in Figure S10, where different combinations of t_e and t_h all satisfying $|t_e + t_h| = 0.03$ eV but with different values of $t_e - t_h$ produce identical absorption spectra while giving rise to distinct densities of states (DOS). In particular, when the hopping integrals have opposite sign, a dense manifold of optically dark states progressively shifts below the lowest bright state. By contrast, when they have the same sign, the lowest bright state is also the first excited state of the system. For a given set of eigenstates, DOS in figure S10 is calculated as:

$$\rho(\omega) = \frac{1}{\mathcal{N}} \sum_i \frac{1}{\sigma\sqrt{2\pi}} e^{-\frac{\omega-\omega_i}{2\sigma^2}} \tag{S11}$$

where $\mathcal{N}$ is the total number of states and $\sigma = 10$ cm^{-1}.

The absorption spectrum alone is therefore insufficient to uniquely parametrize t_e and t_h separately. More information can be extracted from emission. Although experimental studies are only available for the emission of the DWNT, some meaningful consideration is possible. In ref.[14] the presence of a single dark state below the first bright state was hypothesized. On the other hand, out-of-phase t_e and t_h would generate a whole dense manifold of low-lying dark states, highly suppressing the emission of the system. Moreover, the presence of low-lying dark states would be expected to reduce the fluorescence quantum yield upon cooling. Experimentally, however, the opposite behavior is observed, with the quantum yield increasing at lower temperatures.[11] This experimental evidence suggests that the emissive state is therefore the lowest excited state of the system, in turn pointing towards in-phase t_e and t_h.

While t_e and t_h are kept as free parameters in the Hamiltonian, to obtain further indication about their relative sign, we performed TD-DFT calculations (CAM-B3LYP/6-31++G(d,p)) on an optimized dimer of stack 1. This estimate yields $t_e = 0.048$ eV and $t_h = 0.034$ eV. TD-DFT therefore indicates that the two integrals have comparable magnitude and most importantly the same sign, consistently with the conclusions drawn from the available emission data. Values calulated with the software provided in the GitHub repository https://github.com/nicholashestand/exciton_para, copyright ©2017 Nicholas J. Hestand.

References

(1) Deshmukh, A. P.; Zheng, W.; Chuang, C.; Bailey, A. D.; Williams, J. A.; Sletten, E. M.; Egelman, E. H.; Caram, J. R. Near-atomic-resolution structure of J-aggregated helical light-harvesting nanotubes. *Nature chemistry* **2024**, *16*, 800–808.

(2) Hestand, N. J.; Spano, F. C. Interference between Coulombic and CT-mediated couplings in molecular aggregates: H-to J-aggregate transformation in perylene-based π-stacks. *The Journal of chemical physics* **2015**, *143*.

(3) Clark, K. A.; Cone, C. W.; Vanden Bout, D. A. Quantifying the polarization of exciton transitions in double-walled nanotubular J-aggregates. *The Journal of Physical Chemistry C* **2013**, *117*, 26473–26481.

(4) Hestand, N. J.; Spano, F. C. Expanded theory of H-and J-molecular aggregates: the effects of vibronic coupling and intermolecular charge transfer. *Chemical reviews* **2018**, *118*, 7069–7163.

(5) Kriete, B.; Bondarenko, A. S.; Alessandri, R.; Patmanidis, I.; Krasnikov, V. V.; Jansen, T. L.; Marrink, S. J.; Knoester, J.; Pshenichnikov, M. S. Molecular versus excitonic disorder in individual artificial light-harvesting systems. *Journal of the American Chemical Society* **2020**, *142*, 18073–18085.

(6) Kriete, B.; Lüttig, J.; Kunsel, T.; Malŷ, P.; Jansen, T. L.; Knoester, J.; Brixner, T.; Pshenichnikov, M. S. Interplay between structural hierarchy and exciton diffusion in artificial light harvesting. *Nature Communications* **2019**, *10*, 4615.

(7) Kriete, B.; Feenstra, C. J.; Pshenichnikov, M. S. Microfluidic out-of-equilibrium control of molecular nanotubes. *Physical Chemistry Chemical Physics* **2020**, *22*, 10179–10188.

(8) Caram, J. R.; Doria, S.; Eisele, D. M.; Freyria, F. S.; Sinclair, T. S.; Rebentrost, P.; Lloyd, S.; Bawendi, M. G. Room-temperature micron-scale exciton migration in a stabilized emissive molecular aggregate. *Nano letters* **2016**, *16*, 6808–6815.

(9) Pandya, R.; Chen, R. Y.; Cheminal, A.; Thomas, T.; Thampi, A.; Tanoh, A.; Richter, J.; Shivanna, R.; Deschler, F.; Schnedermann, C., et al. Observation of vibronic-coupling-mediated energy transfer in light-harvesting nanotubes stabilized in a solid-state matrix. *The journal of physical chemistry letters* **2018**, *9*, 5604–5611.

(10) Pugžlys, A.; Augulis, R.; Van Loosdrecht, P. H.; Didraga, C.; Malyshev, V.; Knoester, J. Temperature-dependent relaxation of excitons in tubular molecular aggregates: fluorescence decay and Stokes shift. *The Journal of Physical Chemistry B* **2006**, *110*, 20268–20276.

(11) Clark, K. A.; Krueger, E. L.; Vanden Bout, D. A. Temperature-dependent exciton properties of two cylindrical J-aggregates. *The Journal of Physical Chemistry C* **2014**, *118*, 24325–24334.

(12) Bailey, A. D.; Deshmukh, A. P.; Bradbury, N. C.; Pengshung, M.; Atallah, T. L.; Williams, J. A.; Barotov, U.; Neuhauser, D.; Sletten, E. M.; Caram, J. R. Exploring the design of superradiant J-aggregates from amphiphilic monomer units. *Nanoscale* **2023**, *15*, 3841–3849.

(13) Freyria, F. S.; Cordero, J. M.; Caram, J. R.; Doria, S.; Dodin, A.; Chen, Y.; Willard, A. P.; Bawendi, M. G. Near-infrared quantum dot emission enhanced by stabilized self-assembled j-aggregate antennas. *Nano letters* **2017**, *17*, 7665–7674.

(14) Doria, S.; Di Donato, M.; Borrelli, R.; Gelin, M. F.; Caram, J.; Pagliai, M.; Foggi, P.; Lapini, A. Vibronic coherences in light harvesting nanotubes: unravelling the role of dark states. *Journal of Materials Chemistry C* **2022**, *10*, 7216–7226.
